# Severe Radiographic Legg-Calvé-Perthes Disease With Favorable Long-Term Functional Outcomes Following Conservative Management: A Case Report

**DOI:** 10.7759/cureus.102835

**Published:** 2026-02-02

**Authors:** Hannah J Grimmett, Stephen E Martiny

**Affiliations:** 1 Department of Family Medicine, William Carey University College of Osteopathic Medicine, Hattiesburg, USA; 2 Department of Family Medicine, Mercy Health, Cumming, USA

**Keywords:** case report, hip pathology, legg-calvé-perthes disease, pediatric femoral head necrosis, pediatric orthopedics

## Abstract

Legg-Calvé-Perthes disease (LCPD) is a pediatric disorder characterized by avascular necrosis of the femoral head, with prognosis largely determined by the extent of epiphyseal involvement. Severe radiographic classifications are typically associated with poor long-term outcomes and often prompt surgical intervention. We present a case of a male patient diagnosed with unilateral LCPD at age three with extensive femoral head involvement by age six (Catterall Group 4, Salter-Thompson Group B, Herring Group C) who was managed non-operatively over two decades. Treatment consisted of physiotherapy, activity modification, and pain management without surgical intervention. Despite marked femoral head deformity and residual limitations in hip range of motion, the patient achieved high functional capacity, including participation in National Collegiate Athletic Association (NCAA) Division I athletics, and he maintains independence in activities of daily living as a young adult. This case highlights the potential for favorable functional outcomes with conservative management in carefully selected patients with poor-prognosis LCPD and underscores the importance of individualized treatment planning.

## Introduction

Legg-Calvé-Perthes disease (LCPD) is defined as unilateral or bilateral loss of the femoral head as a result of avascular necrosis and has an incidence rate ranging from 0.4/100,000 to 29/100,000 in children younger than 15 years old [1]. It most commonly presents in children ages 3-12, with the highest diagnosis rate in boys ages 5-7; of note, boys are affected 3-5 times more often than girls [2]. Additionally, LCPD presents bilaterally in approximately 10-14% of patients, and there is an identifiable correlation with inheritance in approximately 8-12% of patients [3]. The most common presenting symptoms include hip and knee pain, which are intensified during and after physical activity, decreased range of motion in the hip, and a Trendelenburg gait [1].

LCPD was first described by Arthur Legg, Jacques Calvé, and Georg Perthes in 1909; however, they suspected it to be a result of trauma, abnormal osteogenesis, or inflammation, respectively [4]. While the lack of vascularization has proven to be the most important etiologic factor in the development of LCPD, there have been several theories as to how the blood supply is disrupted, including, but not limited to, obstruction of the superior retinacular artery, lateral epiphyseal artery interruption, and thrombophilia [5-7].

Henning Waldenström described the progression of LCPD via radiographs; the chronologic radiographic stages include the osteonecrosis stage (with ischemia-induced sclerosis), the fragmentation stage (when absorption, fibrovascular invasion, physeal changes, and cartilaginous metaplasia lend a lytic appearance to the epiphysis and metaphysis, with variable collapse), the re-ossification stage (when the fibrovascular and cartilage islands are replaced by bone), and the healed stage (when woven bone is replaced by normal bone) [4]. Because of the varying amount of osteonecrosis of the femoral head, several classification systems have been developed.

In 1971, Catterall described four radiographic groups based on the extent of epiphyseal involvement, ranging from limited anterior involvement with subsequent regeneration (Group 1) to complete epiphyseal sequestration with femoral head flattening and mushroom deformity (Group 4), with progressively worsening prognosis from Group 1 to Group 4 [8]. In 1984, Salter and Thompson proposed a simplified two-group classification based on femoral head involvement of less than 50% (Group A) or greater than 50% (Group B) [9]. In 1992, Herring introduced the lateral pillar classification, which assesses loss of height in the lateral 15-30% of the femoral head during the fragmentation stage, categorizing patients into Groups A (normal height), B (50-100% height), and C (<50% height) [10]. These classification systems were developed to improve prognostic accuracy and guide treatment decisions [11].

This report presents a case of LCPD in a young male following 20 years of conservative management. Successful management was achieved via physiotherapy and pain management, allowing for participation in athletic endeavors. This case highlights the importance of considering both surgical and non-surgical management for patients with poor prognoses in LCPD.

## Case presentation

A 23-year-old Caucasian male presents to his primary care physician for annual follow-up with a past medical history of asthma, attention-deficit hyperactivity disorder (ADHD), dyslexia, and dyscalculia; he was diagnosed with LCPD at the age of three. The patient was born in a breech presentation via an elective C-section, and he reached 39 weeks of gestation with a weight of 4139 g (9 lb 1 oz) at birth to a non-smoking mother. He did not have any hospitalizations resulting from injuries throughout his childhood, but there were several emergency department visits. With respect to fractures, when the patient was 12, he suffered an avulsion of the growth plate in the right elbow; however, this resulted from overuse and pitching while playing baseball. At present, he is a healthy adult with a height of 185.5 cm (73 inches) and a weight of 101 kg (223 lb); he has a heart rate of 82 beats per minute, a respiratory rate of 18 beats per minute, a blood pressure of 118/80 mmHg, and an oxygen saturation of 99%.

**Figure 1:**
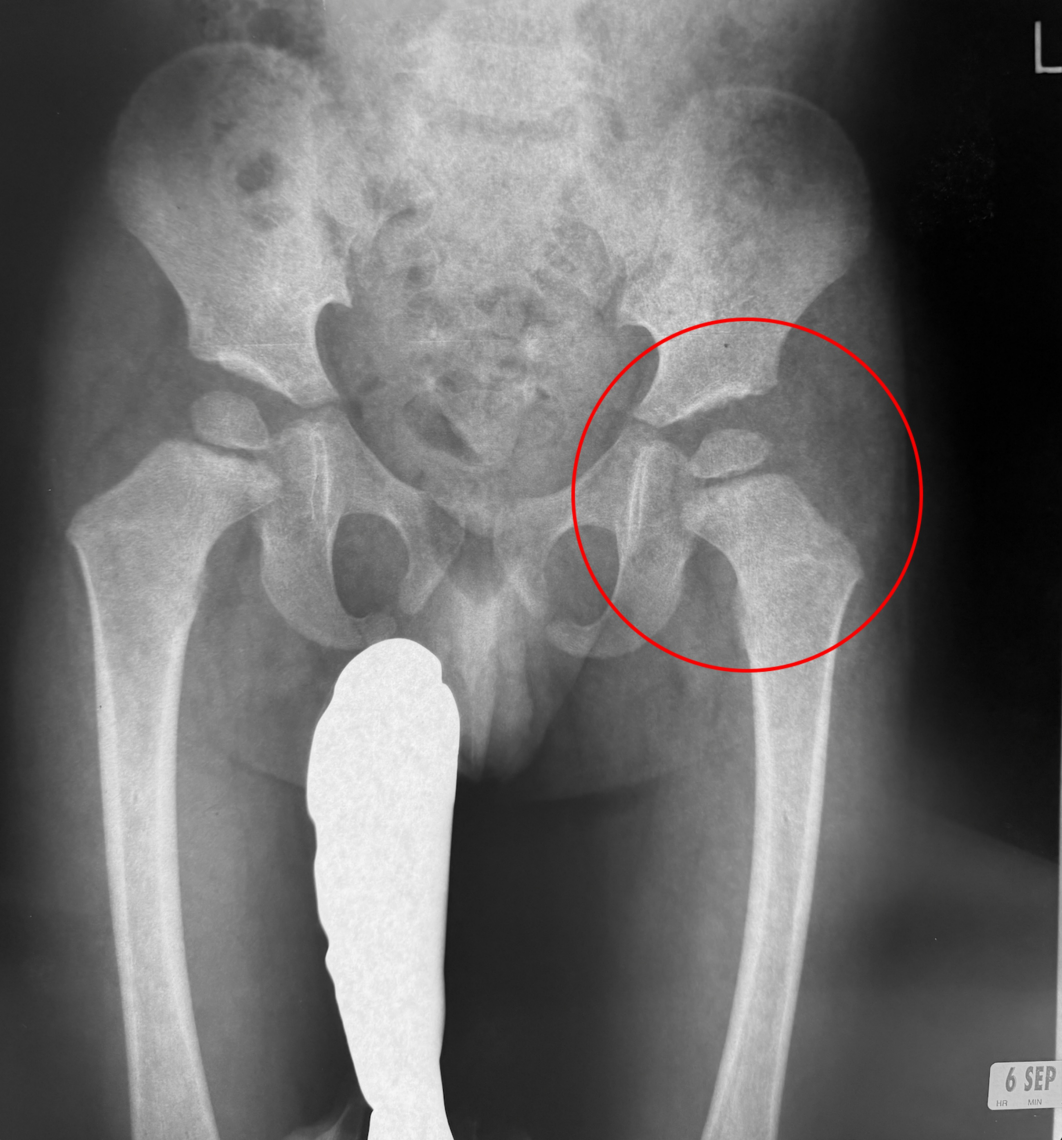
Supine anteroposterior pelvic X-ray taken on September 6, 2005.

With respect to the identification of his LCPD, initially, his pediatrician had a suspicion of the condition based on an X-ray taken after he presented with the complaint of left knee pain and a mild limp in 2005 (Figure 1).

**Figure 2:**
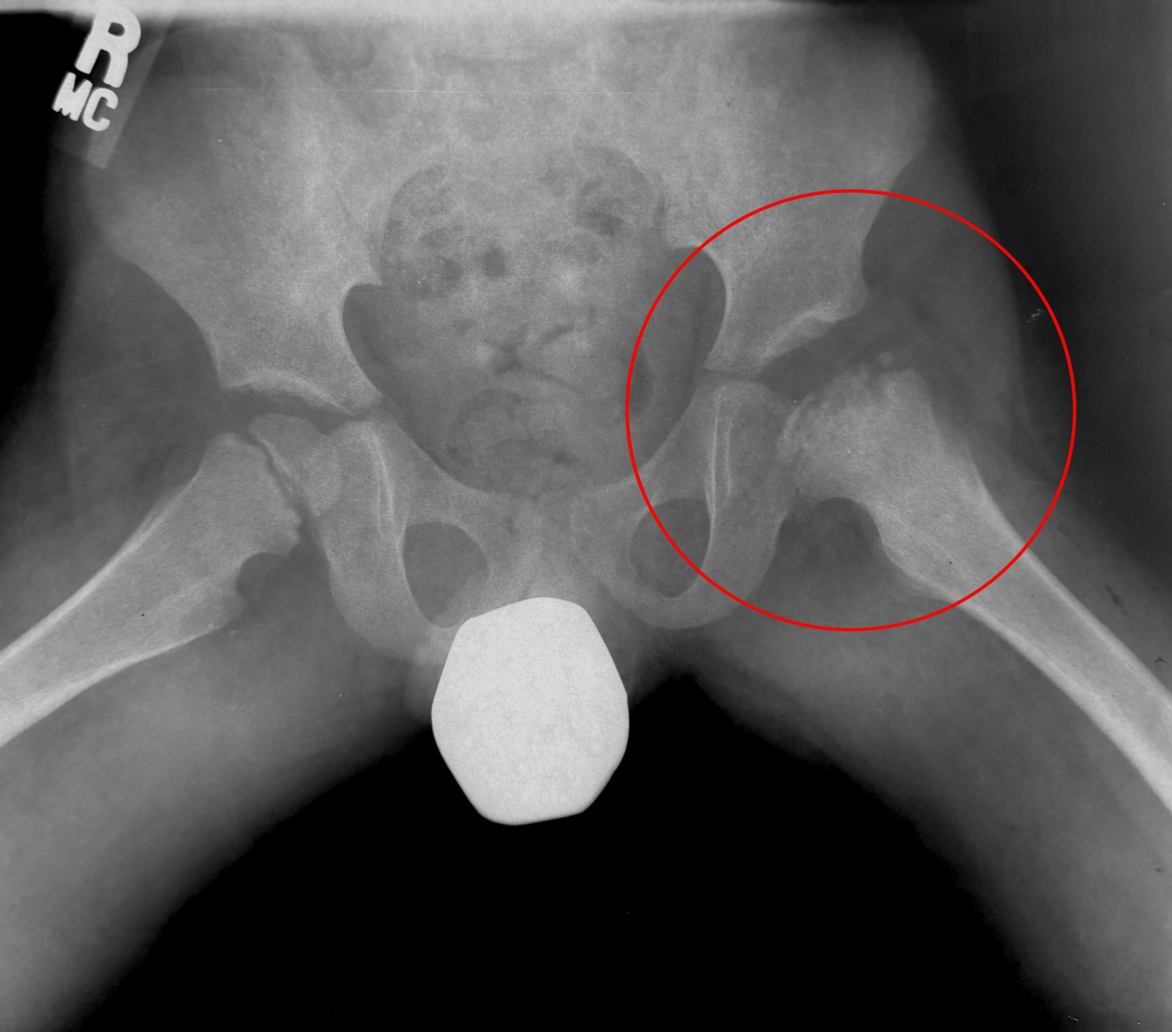
Frog leg lateral view pelvic X-ray taken on March 1, 2007.

He followed up with a pediatric orthopedist who then monitored him for the next several years; during this period, his left femoral head continued to show osteonecrosis and progressed into the fragmentation phase (Figure 2).

**Figure 3:**
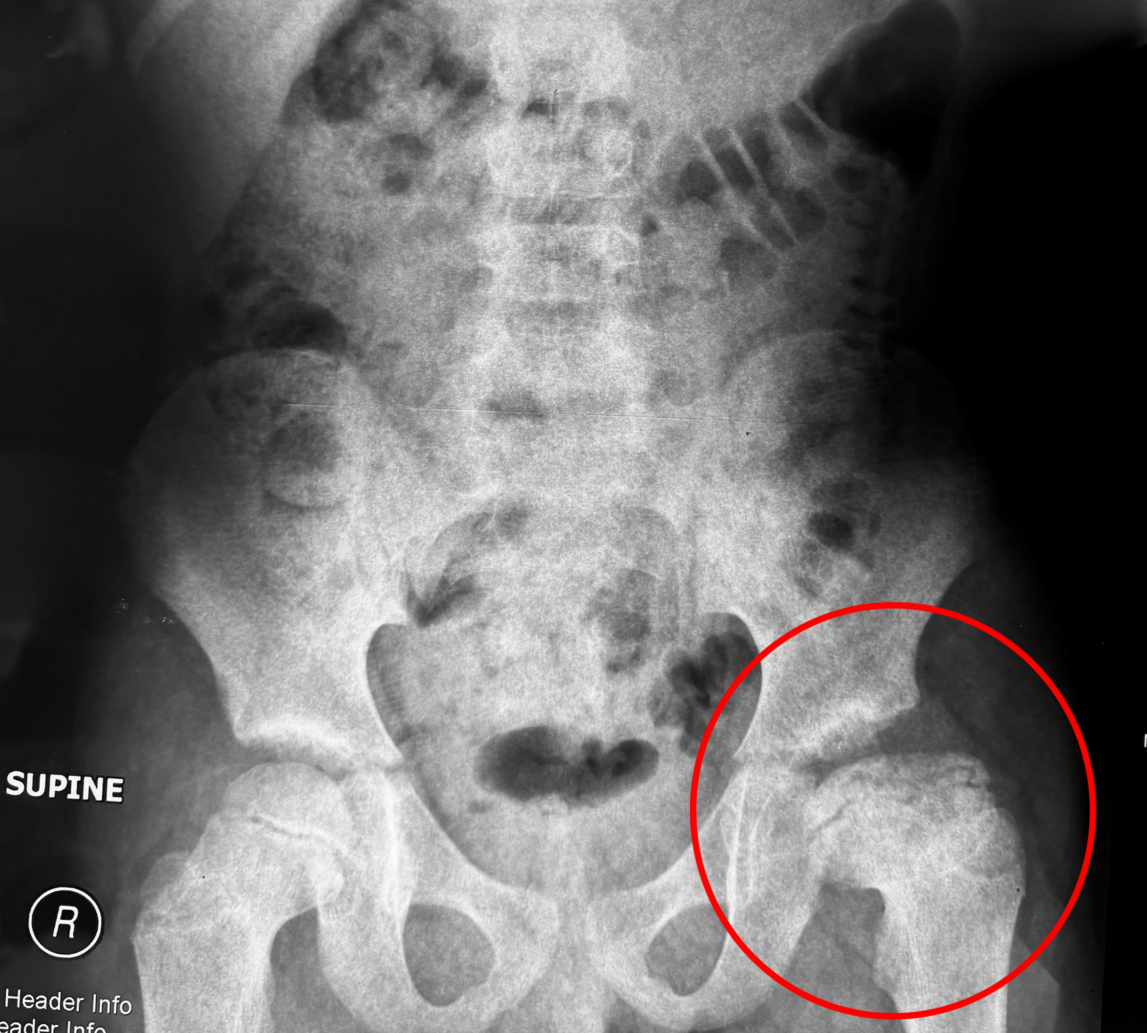
Supine anteroposterior pelvic X-ray taken on December 28, 2009.

**Figure 4:**
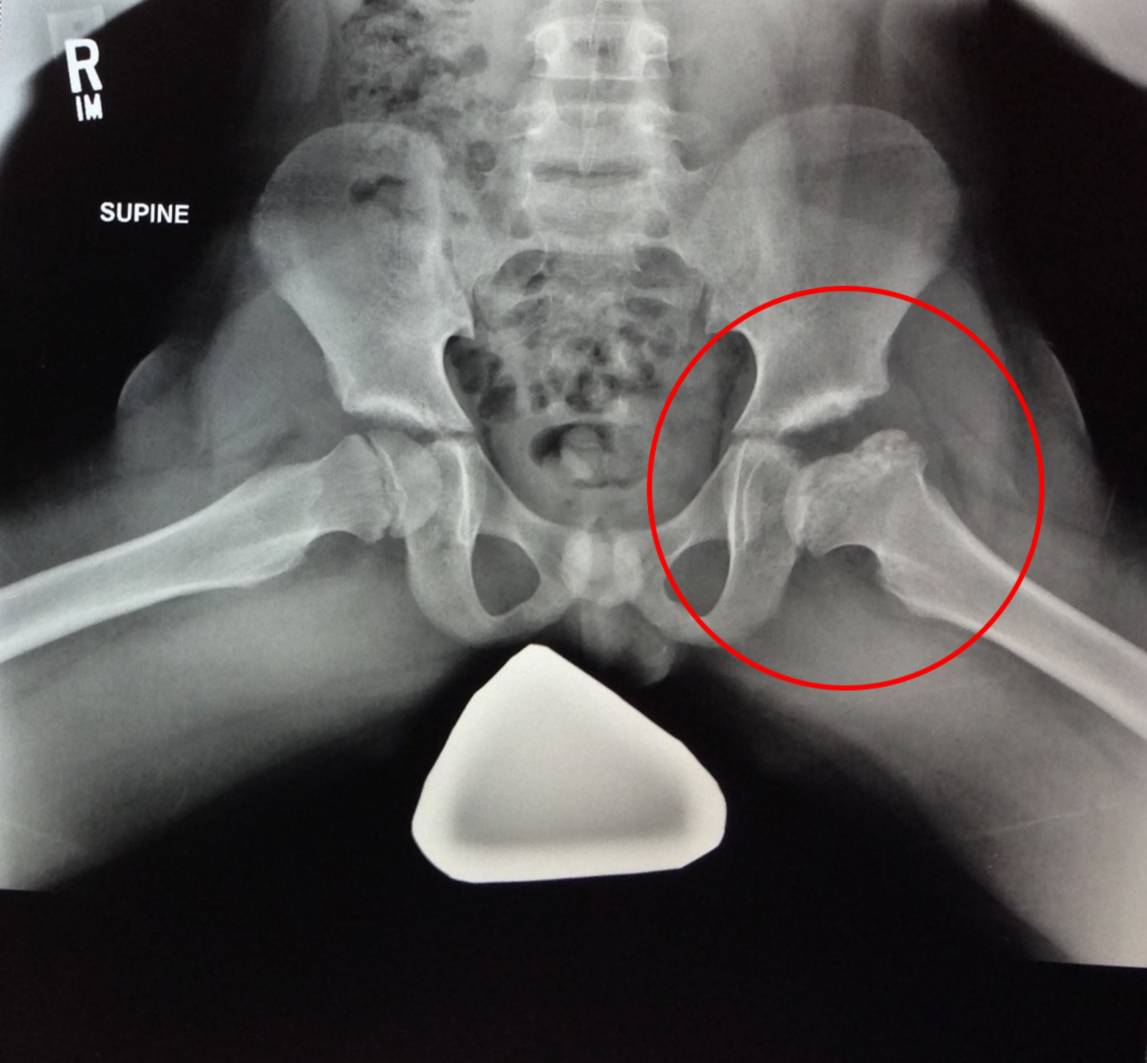
Frog leg lateral view pelvic X-ray taken on December 21, 2011.

The severity of his LCPD, with respect to the classification systems, had him considered to be Catterall Group 4, Salter and Thompson Group B, and Herring Group C; he had total cavity loss of nearly 100% of the area, maximum subchondral damage, and over 50% damage to the lateral abutment. Over time, he progressed through the fragmentation phase and continued to the re-ossification stage (Figures 3, 4).

**Figure 5:**
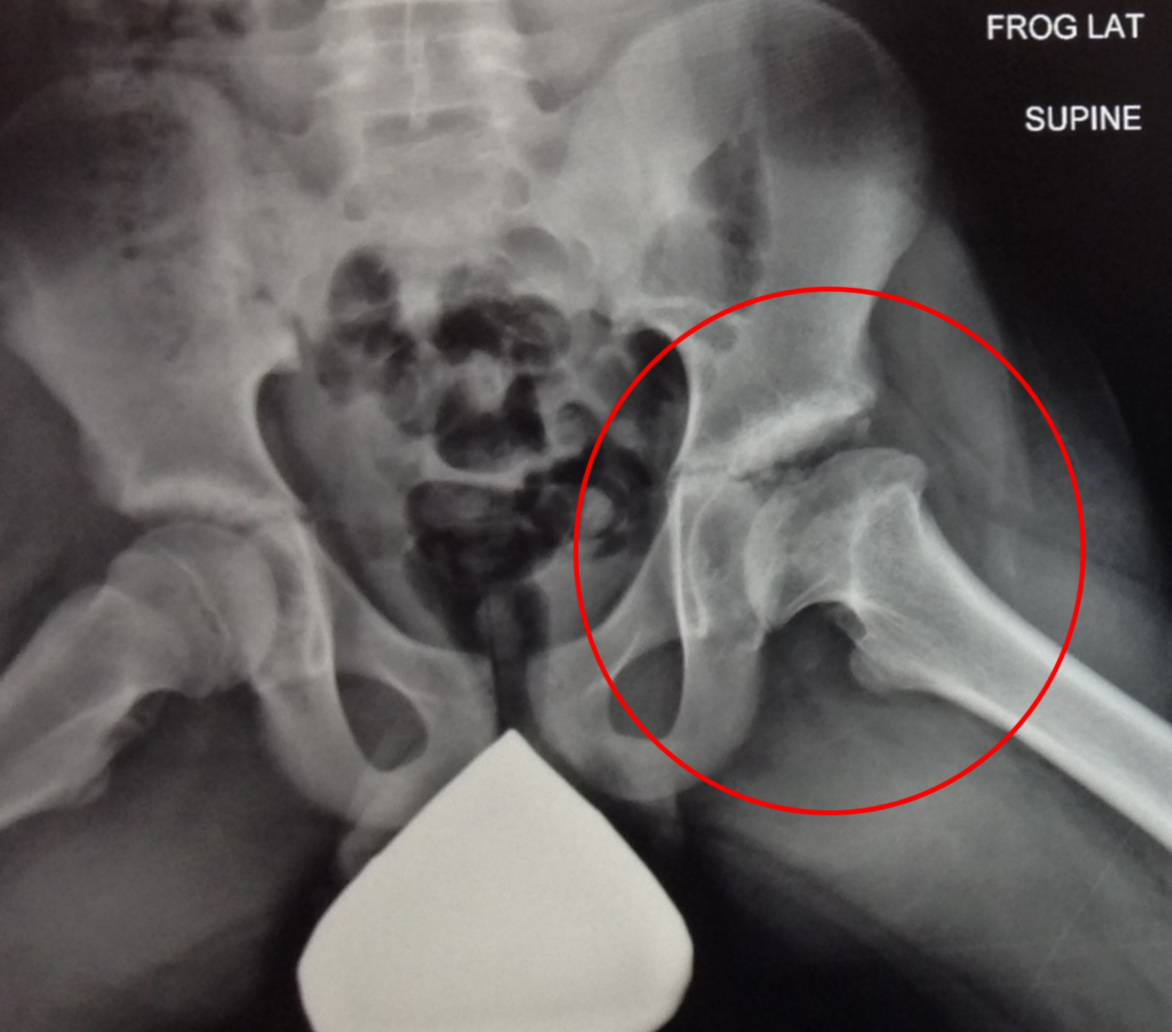
Frog leg lateral view pelvic X-ray taken on January 6, 2016.

**Figure 6:**
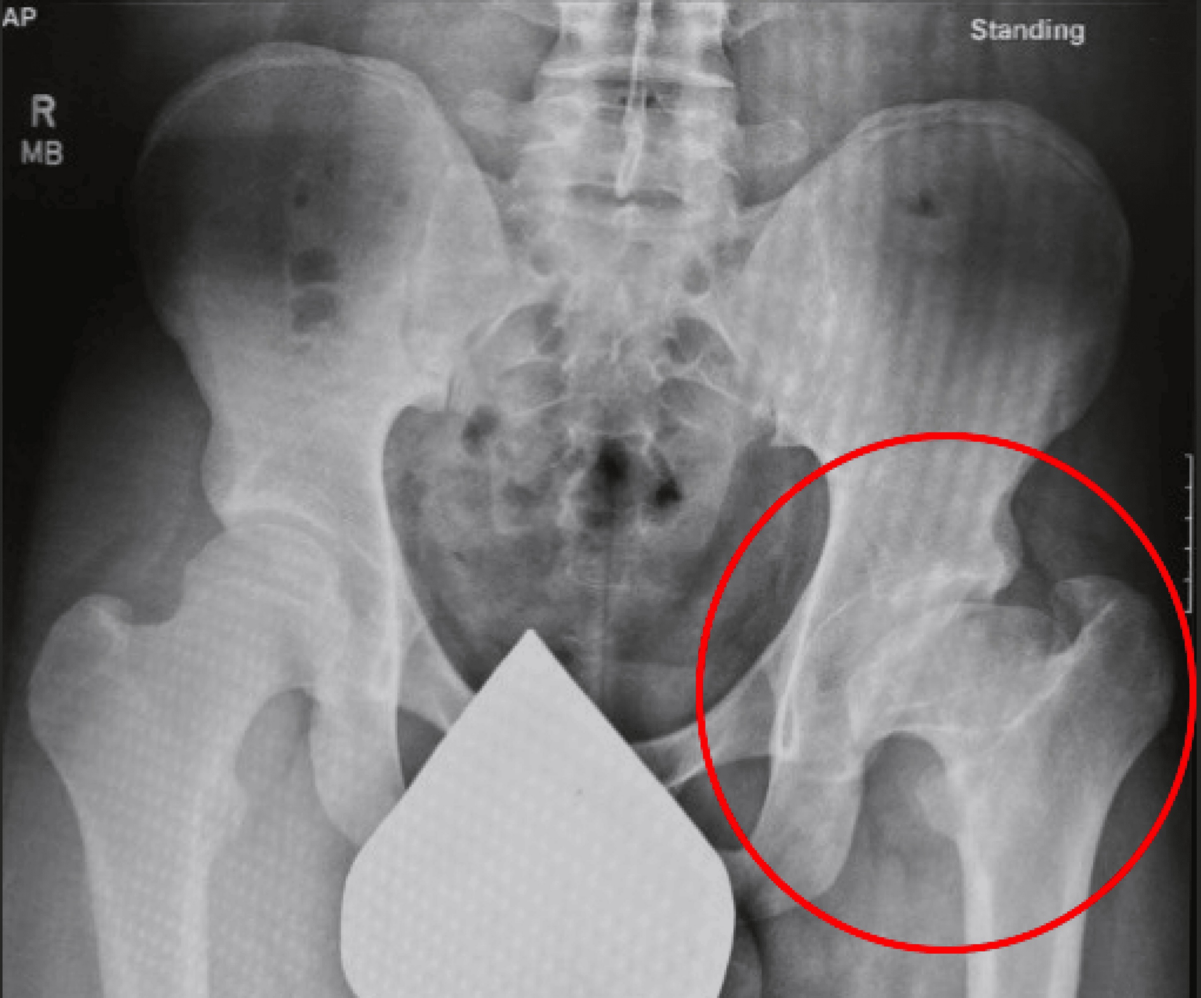
Standing anteroposterior pelvic X-ray taken on December 4, 2019.

At the age of 11, the patient underwent evaluation by a pediatric orthopedist. The orthopedist recommended conservative management over surgery based on observations that valgus osteotomies often led to osteoarthritis and eventually required hip replacements. The patient opted for non-surgical management and continued with activity-based physiotherapy that included rock climbing, horseback riding, and swimming to help maximize his range of motion and limit the concussive force placed on the joint. By the year 2016, the patient had reached the imaging criteria for the healing stage (Figures 5, 6).

Despite his poor prognosis, the patient was able to participate in a National Collegiate Athletic Association (NCAA) Division I-level baseball program, where his maximum deadlift was 249.5 kg (550 lb) and his maximum squat was 183.7 kg (405 lb). He participated in all athletic activities and workouts with no modifications while utilizing nonsteroidal anti-inflammatories (NSAIDs) for pain management.

The patient has a mild leg length discrepancy of 1.25 cm (0.5 inch) in addition to a 7.7 cm (2.88 inches) difference in thigh circumference measured 15.25 cm (6 inches) from the inseam; additionally, his Harris Hip Score is 82.85%, thus indicating a good functional outcome. Regarding quality of life, the patient reports hip pain after walking a substantial distance or remaining stationary for a long period of time, and he ambulates with a slight limp whenever he is not wearing the 1.25 cm (0.5 inch) lift in his left shoe. Pain management consists of NSAIDs as needed. Additionally, the patient reports no limitations to activities of daily living; however, goniometer measures do show decreased range of motion of the left lower extremity and bilaterally decreased flexibility of the lower extremities that might warrant hip replacement in the next five years (Table 1).

**Table 1:** Hip range of motion measured in degrees via a goniometer. Hip flexion, abduction, and adduction were recorded with the patient in a supine position; hip extension was recorded with the patient in a prone position; and hip internal and external rotation were recorded with the patient in a seated position with 90° of knee flexion.

Range of Motion	Right Leg	Left Leg	Normal Range
Flexion	115	95	110–120°
Extension	30	30	20–30°
Abduction	38	30	30–50°
Adduction	21	15	20–30°
Internal Rotation	33	27	30–40°
External Rotation	52	41	40–60°

## Discussion

As of now, there is no proven underlying cause for LCPD; however, several associations with the condition have been identified, including maternal smoking, low birth weight (<2499 g), breech presentation, and both preterm and post-term births [12,13]. It is important to note that maternal smoking is also associated with low birth weight [14]. As a result, it is difficult to conclude whether or not LCPD is associated with low birth weight independently, or if maternal smoking contributed to the low birth weight [12]. The risk of developing LCPD was up to 25% higher in patients who were born breech. Lindblad et al. speculated that the increased pressure on the hip joint might contribute to the compromise of vascularization. Additionally, LCPD was also associated with very preterm (<32 weeks), preterm (32-36 weeks), and post-term (>42 weeks) deliveries [13]. In this case, no maternal smoking was documented, and the patient did not have a low birth weight; however, the patient was breech.

With respect to psychological development, Hailer and Nilsson noted the association between ADHD and LCPD [15]. Additionally, Hailer et al. found that LCPD was significantly associated with increased risk of injuries requiring hospitalization. LCPD patients had a 26% higher risk for soft tissue injuries and a 14% higher risk for fractures. They speculated that the increased risk of injury might result from decreased muscle strength, derangement of joint mobility, coordination problems, or bone mineral loss due to immobility caused by LCPD [16]. However, it is important to note that it has also been speculated that the presence of ADHD increases risk-taking behaviors, thus resulting in an increased number of injuries requiring hospitalization [15]. Although the patient, in this case, has a diagnosis of ADHD and experienced emergency room visits related to risk-taking behaviors, no injuries required hospitalization.

Asthma is an additional diagnosis that could potentially be associated with LCPD; interleukin-6 (IL-6) is strongly associated with the pathophysiology of asthma, and it has also been found significantly elevated in LCPD patients [12,17]. IL-6 is a cytokine with pleiotropic activity that can lead to the onset or development of various diseases; it induces the synthesis of acute-phase proteins such as CRP, serum amyloid A, fibrinogen, and hepcidin in hepatocytes, whereas it inhibits the production of albumin [18]. The patient identified in this case acquired an asthma diagnosis at a young age, as he suffered from an asthma attack warranting an emergency room visit as a child. While his serum IL-6 levels have never been checked, it would be reasonable to hypothesize that, because of his diagnoses of both LCPD and asthma, he could also have elevated IL-6 levels.

Based on this patient’s Catterall (Group 4), Salter-Thompson (Group B), and Herring classifications (Group C), determined as the disease progressed, his LCPD carried a notably poor prognosis despite early diagnosis. Management strategies for severe LCPD remain heterogeneous, ranging from early surgical intervention to prolonged conservative treatment and physiotherapy [19]. Currently, there is minimal available literature discussing the long-term results of either conservative management or surgical management of LCPD patients who were diagnosed very early in life. As a result, this case contributes to the existing literature on non-operative management by demonstrating that, in select patients, conservative treatment may support meaningful functional outcomes. Notably, this individual was able to participate in high-level athletics throughout adolescence and early adulthood despite significant femoral head deformity, underscoring the potential role of individualized, non-surgical approaches even in cases traditionally associated with unfavorable prognoses.

## Conclusions

This case demonstrates that select patients with severe, poor-prognosis LCPD may achieve sustained functional independence and even high-level athletic participation into early adulthood with prolonged conservative management. Despite radiographic features traditionally associated with unfavorable outcomes, including complete epiphyseal involvement, femoral head deformity, and lateral pillar collapse, non-operative treatment focused on physiotherapy, activity modification, and symptom-guided pain management resulted in preserved function and favorable Harris Hip Score outcomes, delaying surgical intervention.

While this outcome should not be interpreted as representative of all patients with severe LCPD, it highlights the limitations of relying solely on radiographic classification systems to predict long-term functional capacity. This case supports individualized treatment planning that integrates patient goals, functional demands, symptom burden, and validated functional outcome measures alongside imaging findings.
